# Supplementary material for: Nondestructive real-space imaging of energy dissipation distributions in randomly networked conductive nanomaterials
Source: Sci Rep. 2019 Oct 10;9:14572. doi: 10.1038/s41598-019-50802-z (PMC6787255; doi:10.1038/s41598-019-50802-z)
Supplement: Supplementary file 1 — Supplementary Information [file 41598_2019_50802_MOESM1_ESM.pdf]

Supplementary information

Nondestructive real-space imaging of energy dissipation distributions in randomly networked conductive nanomaterials

Takahiro Morimoto, Seisuke Ata, Takeo Yamada and Toshiya Okazaki**

CNT-Application Research Center, National Institute of Advanced Industrial Science and Technology (AIST), Tsukuba 305-8565, Japan

S-1. The fundamental process of LIT method

In this manuscript, we have applied the real-time lock-in thermography (LIT) technique to measure the current density distribution and randomly networked structures via the Joule heating from the sample surface. This method is basically similar to the "dual phase lock-in technique" in conventional electric or optical measurement systems. In the conventional systems, the lock-in process is applied for improving the signal noise ratio (S/N). In our case, the LIT method is mainly used as frequency space separations of various heat components.

In this time, we used the THERMOS-1000 (Hamamatsu Photonics) as base setup of the lock-in thermography (LIT) systems. The IR camera is consisted with an InSb detector cooled by a Stirling cooler. The maximum pixel size is (640 x 512) at 25 Hz measurement. The IV characteristic and ac bias voltage are applied by the semiconductor parameter analyzer (B1500, Keysight Technologies). The detected IR wavelength was ranged from 3.0 to 5.0 μm. This range is suitable at room temperature measurements from the estimation based on the Stefan-Boltzmann's law.

The Joule heating is generated by applied bias voltages with rectangular 25 Hz repeated signals. The original data is obtained as IR images in each frame. These IR images provide the thermal intensity depending on the ac bias voltages. In the real time LIT systems, the lock-in calculation is done in each cycle step. After calculation in each step, the original IR frame data is removed from the PC memories for avoiding the huge data creations for long time accumulations. By this system, we can select the suitable

accumulation time beyond limitation of the storage and memory capacity of PC systems. Then, finally, we obtain the intensity and phase images constructed by calculated all pixel data.

Figure S1 shows the schematic picture of the calculation process in this method. The intensity R having phase θ is estimated complex vector 0° and 90° as shown in Fig. S1. From this process, the intensity R is always calculated without phase matching process. This is similar to the dual phase lock-in technique in conventional method. Actually, the heat cycle line-shape is lost by this process. The averaged phase value is also calculated, and this is related to the delay of heating cycles.

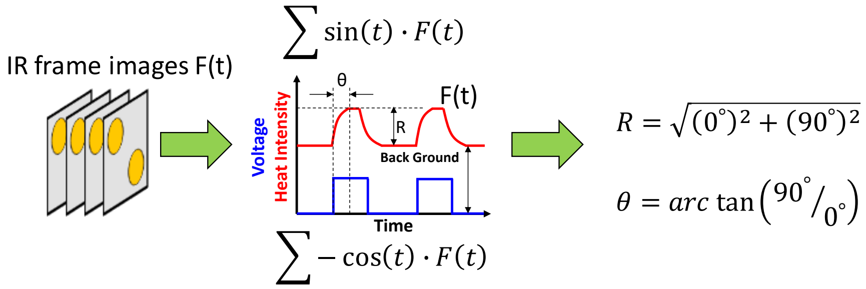

Fig. S1 Schematic picture of the LIT method process

The schematic picture of the LIT method. The raw data is observed as IR images with IR camera systems. The intensity and phase parameters are corresponding the vector length and angle in the complex coordinate space.

S2. The phase separation images in the thin sample.

The difference of heating phase is clearly visualized by the mixing of intensity and phase in each pixel. Figure. S2 shows the results of different timing image calculated as $I_{LIT} = R\,cos\,\theta$. In the low frequency ac bias conditions, the ac current is immediately excited and generate the Joule heating at the current path. On the other hand, the non-active region without excited currents has delay heat component due to the heat conduction from the heat source. Therefore, the faster on-phase component indicated the real current density distributions. The disappeared region in the on-phase image are observed in delay component images. These regions have heat component having same frequency of the ac bias voltages. This expectation is confirmed by the close-up images as shown in Fig. S2b - 2d. In this image, the broken CNT is observed, and this CNT's signal is completely suppressed in the delay component image. This is naturally understood that the current path should be interrupted at the broken point. In these non-active region, the LIT intensity contained only the thermal conductive component without a Joule heating source. Therefore, we compared the LIT intensity and theoretical estimated curves in

these regions.

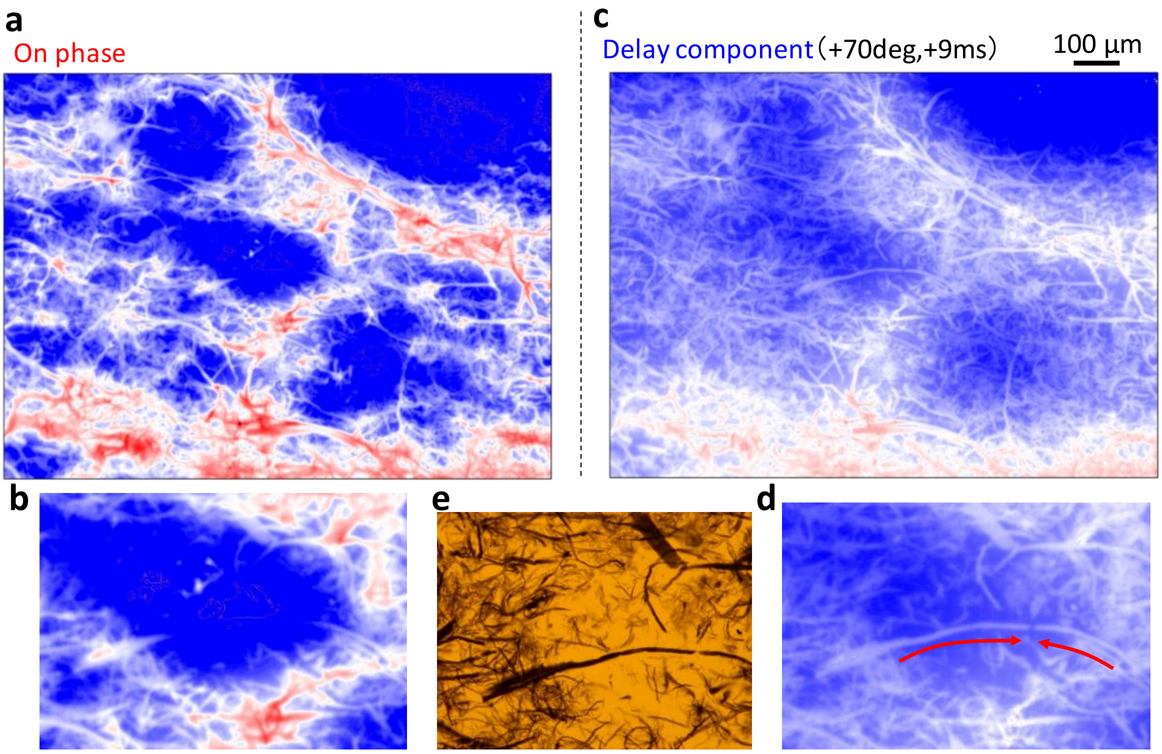

Fig. S2 The phase separation images in the thermally thin sample

The phase separation LIT images in higher magnification measurement. (a) and (b), The on-phase component images having same frequency and phase with an ac bias voltage. (c) and (d), The delay component images. In these images, the thermal conductive signals were observed existed around the Joule heating source area. (e), The close-up image by optical microscope at same position of (b) and (d).

S3. The theoretical estimation based on the heat conduction in thermally thin samples.

In the LIT method, the temperature distributions are observed via the heat radiation at the sample surface. These temperature differences are originated from the Joule heating and thermal conduction to the environment from the heat source. Therefore, the intensity of LIT images should be related to the thermal conduction in microscopic pictures. Especially, in the ac bias conditions, the heat and temperature also have oscillating time dependent components.

The temperature dumping of heat conduction with oscillation heat source is represented as:

$$\Lambda = \sqrt{\frac{2\alpha}{f}}$$

here, Λ is the thermal diffusive length, α is the thermal diffusivity and f is the frequency. The intensity of conductive temperature is decreased as $1/e$ at this thermal diffusive length. For estimation of the LIT intensity, the sample thickness is one important factor for thermally thickness. In the case of thermally thin sample (sample thickness is thinner than the Λ), the thermal conduction should be occurred including multiple reflections at the interfaces. These multiple reflections are considered as the mirror heat source. Therefore, the point like heat source should act as cylindrical-shape heat sources. In these descriptions, the thermal conduction should be described based on the one kind of the Bessel functions.

$$T(r,t) = AK_0\left(r\sqrt{\frac{iC_P n\omega}{\lambda}}\right)e^{i\omega t} = A\left(ker\left(\frac{r\sqrt{2}}{\Lambda}\right) + ikei\left(\frac{r\sqrt{2}}{\Lambda}\right)\right)e^{i\omega t}$$

here is, C_P is specific heat, λ is thermal conductivity, n is material density and r is distance from the heat source. Figure S4 shows the estimated curves of various typical materials. The all curve shows strongly intensity dumping near the heat source. This strongly dumping curve is important for higher resolutions of the LIT method. This result suggests that the LIT method has high spatial resolution between a heat source and non-active regions.

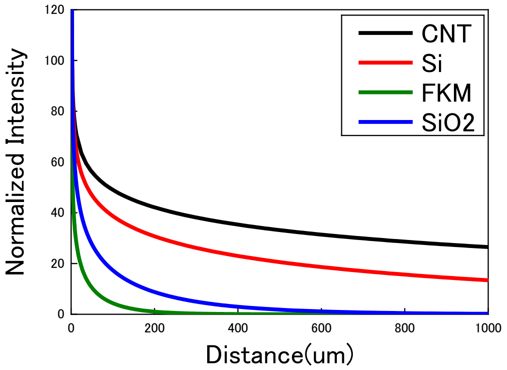

Fig. S3 The estimated theoretical curves of various typical materials

The thermal dumping curves calculated by the thermal conduction equation. Each carve is corresponding to the typical material, single CNT (black), silicon (red), silicon di-oxide (blue) and FKM rubber (green). The CNT bundle had similar thermal conductivity value with the silicon.

S4. The quantitative analysis of LIT intensity distributions

The LIT intensity distributions should be depended to the homogeneity of CNT network structures. By increasing the CNT concentrations, the uniform network structures are easily realized based on the percolation models. As shown in Fig. S4, the lower density sample indicated strongly asymmetric line-shape. Especially, the long tail line-shape is caused by the bottleneck structures as shown in Fig. 2. In this case, it is difficult to fit the curve by the normal distribution function as shown in Fig. S4**a**. On the other hand, the higher density sample shows almost completely normal distributions. The full-width and half maximum (FWHM) is also smaller than the lower density samples.

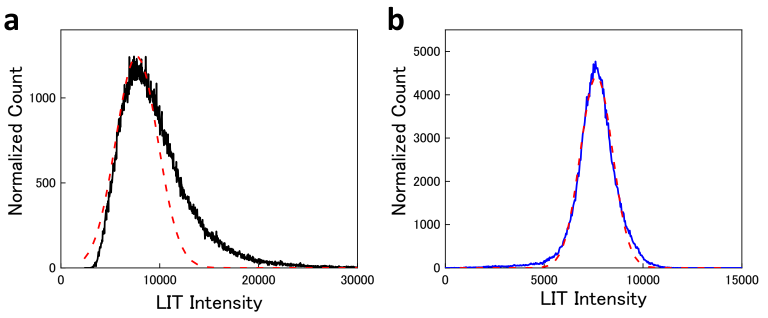

Fig. S4 The LIT intensity distribution and normalized distribution fitting curves

(a) and (b), The LIT intensity distributions are shown with the normalized distribution fitting curves. The higher density sample (1.0 wt%) had almost perfect fitting with normal distributions as shown in (b). The lower density sample (0.25 wt%) had asymmetric distributions due to additional higher intensity components.

S5. The current flow direction dependence in same network structures.

In the case of the randomly connected network structures, the current path should be chosen for minimize the total impedance between electrodes. These electric circuits are consisted with the nano-material's resistance and junctions between nano-materials. Therefore, it is possible that the local low resistance region is not effective in different bias configurations.

Figure. S5 shows the current flow direction dependence observed in same sample and measurement area. In the cross-shape sample, the non-active regions are clearly observed depending on the current flow directions as shown in Fig. S5a and 5c. These results showed that the non-active region does not contribute to reduce the total impedance by their long current path. This simple rule is also effective in higher magnification measurement for a few μm scale images. For example, in the black circles potion, the strong LIT intensity is observed in horizontal current conditions. In the case of vertical current flow conditions, however, the current path is completely different and avoided in

this region. This difference should be originated from the difference of orientation and connectivity of CNTs. These results showed that the LIT method have possibility to analyze the detail of transport mechanism in these percolated network structures.

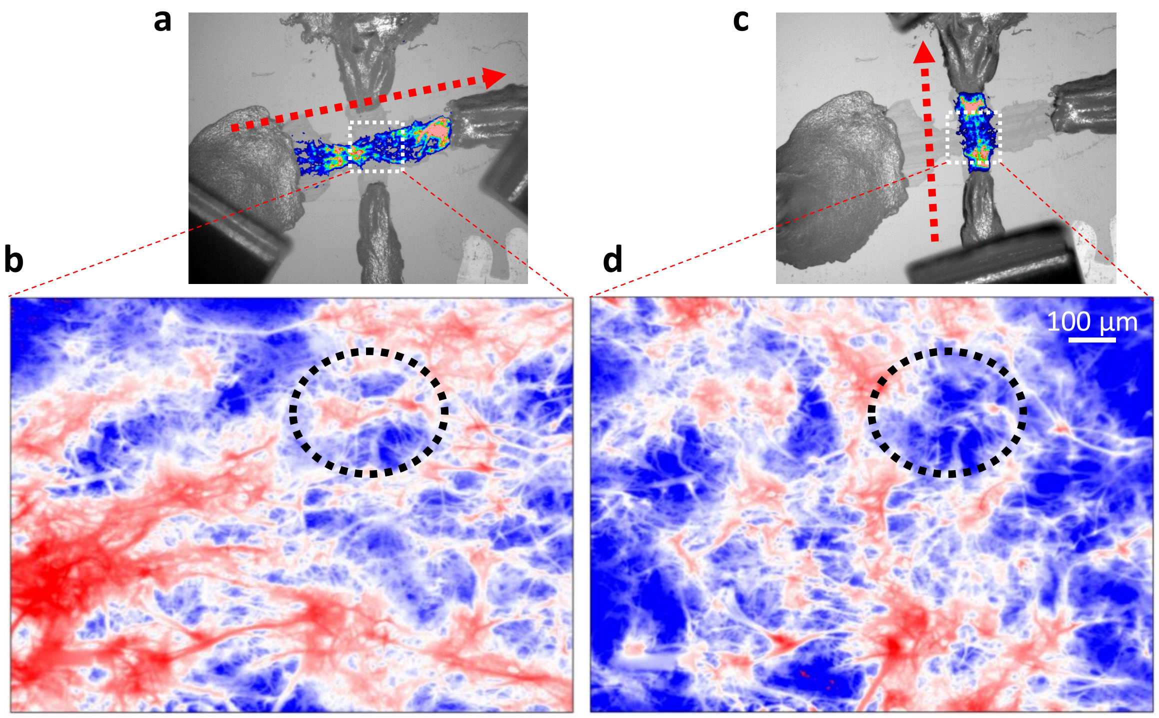

Fig.S5 The current flow direction dependent LIT images on same sample and area

(a) and (c), The overlay images of LIT and IR image observed by different current flow direction. (b) and (d), The high magnification LIT images in each current flow direction. The black dot circles indicate the same potion.
